# Association between short-term exposure to sulfur dioxide and carbon monoxide and ischemic heart disease and non-accidental death in Changsha city, China

**DOI:** 10.1371/journal.pone.0251108

**Published:** 2021-05-03

**Authors:** Zenghui Xu, Lili Xiong, Donghui Jin, Jie Tan

**Affiliations:** 1 Changsha Environment Protection College, Changsha, Hunan, China; 2 Hunan Province Maternal and Children Care Hospital, Changsha, Hunan, China; 3 Centre for Disease Control and Prevention of Hunan Province, Changsha, Hunan, China; 4 Hunan Province Environmental Monitoring Centre, Changsha, Hunan, China; The Ohio State University, UNITED STATES

## Abstract

**Background:**

To investigate the effects of short-term exposure to sulfur dioxide (SO_2_) and carbon monoxide (CO) in the central and southern China areas on ischemic heart disease (IHD) and non-accidental death*s*.

**Method:**

We investigated the associations between short-term exposure to SO_2_ and CO in a city in south-central China and IHD and non-accidental death using a time-series design and generalized additive models with up to a 5-day lag adjusting for day of the week, temperature, air pressure, wind speed, and relative humidity. The relative risks of IHD and non-accidental death per 10-unit increase in SO_2_ and CO were derived from zero to five days in single-pollutant models.

**Results:**

Between 2016 and 2018, a total of 10,507 IHD and 44,070 non-accidental deaths were identified. The largest significant relative risk for IHD death was lag 02 for both SO_2_ (1.080; 95% confidence interval: 1.075–1.084) and CO (5.297; 95% confidence interval: 5.177–5.418) in single-pollutants models. A significant association was shown at all lag multiple-day moving averages. Two-pollutant models identified an association between SO_2_ and mortality when adjusting for CO. In stratified analyses, SO_2_ exhibited a stronger association with death during the cold season, while CO exhibited a stronger association with mortality from IHD during the warm season. The risk of death was more robust in the elderly for both pollutants, but was greater in men for CO and in women for SO_2_.

**Conclusions:**

Overall, we found an association between short-term exposure to low-level SO_2_ and CO and the risk of IHD and non-accidental death.

## 1. Introduction

Globally, estimated years of life lost from ischemic heart disease (IHD) increased by 20.9% (19.0–22.9) between 1990 and 2007, and by a further 17.3% (15.4–19.0) between 2007 and 2017 [1]. IHD has become the second leading cause of death in China [2]; various clinical, epidemiological, and toxicological studies have provided evidence that ambient air pollution likely contributes to the development and exacerbation of IHD [3–6]. Ambient air pollution causes the deaths of an estimated 4.2 million individuals each year [7], and IHD accounts for 25% of deaths related to air pollution [8]. Sulfur dioxide (SO_2_) and carbon monoxide (CO) have both been associated with a higher risk of heart failure and death [9–11].

SO_2_ exerts adverse health effects through systemic inflammation and oxidative stress [12, 13]; in systemic circulation, SO_2_ can enhance the risk of cardiorespiratory mortality and morbidity [14], coronary heart disease [15], and IHD [14, 16]. However, most research in developing nations has focused on particulate matter and not on SO_2_ pollution. China is the leading SO_2_ emitter in the world, with the average for annual-mean SO_2_ levels in 338 Chinese cites reported as 3–87 μg/m^3^ in 2015 [17], well beyond the ranges in developed countries [18]. However, with the efforts made by the Chinese government in recent years to improve the ecological environment, SO_2_ pollution has been largely mitigated and the average annual-mean SO_2_ levels in 338 Chinese cites reported as 13 and 11 μg/m^3^ in 2018 and 2019, respectively [19]. Is SO_2_ at lower concentrations associated with the deaths in China? The problem is worthy of exploring.

Ambient CO could cause myocardial ischemia and rhythm disturbance at lower concentrations [20], increase the number of strokes and enhance the risk of IHD mortality at short-term exposure [11, 21]. As the largest developing country in the world, China is undergoing unprecedented advances in urban motorization. As a result, ambient CO pollution has become a serious environmental issue in many cities in China where the number of motor vehicles has increased rapidly over the past few decades. As we all known, the incomplete combustion of fossil fuels in motor vehicles was mainly producing CO. However, compared with the ample evidence supporting the coherence and plausibility of the association between CO and mortality in the USA and Europe [22, 23], few studies have explored these associations in China, where air pollution characteristics, meteorological conditions, and socioeconomic patterns are different from those developed countries.

Non-accidental deaths consistent with the law of life or disease development, is considerable to estimate the association between short-term exposure to air pollutants and daily deaths, and is often used as the compare group with the disease being studied [24]. Although epidemiological studies in developed nations have provided evidence of an association between ambient SO_2_ and CO pollution and mortality, fewer studies have been conducted on this topic in China. Thus, there is a pressing need to evaluate the association between the current exposure level of SO_2_/CO and mortality from IHD. The present study was a time-series analysis of the effects of short-term exposure to SO_2_ and CO in the urban districts of Changsha city in southern-central China on IHD and non-accidental deaths during the period of 2016 to 2018, to provide evidence for exploring the mortality risks associated with low concentrations exposure of SO_2_ and CO in China.

## 2. Data and methods

### 2.1. Study area

**Fig 1:**
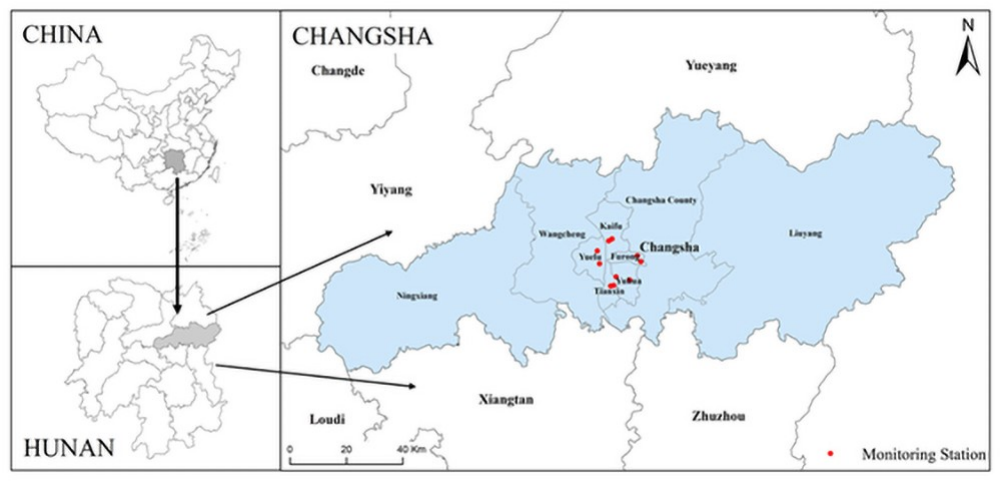
District map of Changsha, Hunan Province in China, with the locations of air quality monitoring stations. The map was created by the Arcgis10.6 software. This figure previously appeared in https://doi.org/10.1016/j.apr.2021.01.022..

Changsha is the capital city of Hunan Province (28°12′N, 112°59′E), with an area of 11,819 km^2^ and a population of 7.9 million. Five urban districts (Furong, Kaifu, Tianxin, Yuelu, and Yuhua), are included in Changsha, totaling an area of approximately 1,216 km^2^ (Fig 1). The population was approximately 3.5 million in 2019.

### 2.2. Daily death counts, air pollution, and meteorological data

The personal information of deaths between January 1, 2016 and December 31, 2018 were obtained from the National Mortality Surveillance System, operated by the Center for Disease Control and Prevention of Hunan province. The information of each death was including birth and death dates, sex, age, cause of death and other demographic factors. Causes of death were assigned from death certificates and were coded by the International Classification of Diseases 10th Edition (ICD10). We extracted and analyzed the information of deaths during the study period and districts by searching I20–I25 for IHD and A00-R99 for non-accidental deaths with the ICD-10 codes.

The air pollution data during the study period were collected by 10 monitoring stations in the five urban districts of Changsha and provided by the Changsha Environmental Protection Bureau. The concentrations of daily 24-h average SO_2_ (μg/m^3^) and CO (mg/m^3^) were used as the exposure. The concentrations of SO_2_ and CO included in the model were the average of the measurements of the 10 monitoring stations. These stations were consistent with the site selection requirements of national air monitoring stations, and they were located away from industrial sources, major roads, or residential sources of emissions including coal, waste, or oil. Thus, our results reflect the background urban air pollution level in Changsha rather than local sources of pollution such as traffic or industrial combustion [25]. K-nearest neighbor imputation for the outliers and few missing values of the air pollution data was implemented, and it was replaced by the average of the 3 days before and after [26].

Meteorological data, including temperature, air pressure, wind speed, and relative humidity during the study period were obtained from Changsha Meteorological Bureau. There were no missing data.

The study protocol was approved by the Ethic Review Committee of Changsha Environment Protection College and carried out in accordance with the principles of the Declaration of Helsinki. All data on patient death records was fully anonymized prior to the researchers accessing them.

### 2.3. Statistical analysis

Mean ± standard deviation and quartile were calculated for the descriptive analysis of variables. Spearman’s rank correlation was used to evaluate the association between meteorological factors and air pollution. Daily data for concentrations of SO_2_ and CO and weather conditions were pooled together for the same time to match the daily IHD and non-accidental deaths. A longitudinal time-series design was conducted to evaluate the associations between the short-term expose to SO_2_/CO and daily IHD and non-accidental deaths. A quasi-Poisson regression (quasi-likelihood) in generalized additive models was used to estimate the association between short-term expose to SO_2_ and CO and daily IHD and non-accidental deaths. Smoothing spline functions were applied to control the effects of confounding factors such as secular trends, meteorological factors, and day of the week.

We used the model Log[E(Y_t_)] = α+DOM+βX_t_ + s (time, df)+s (Z_t_, df), where t is the observation date; Y_t_ is the daily death count on day t, E(Y_t_) is the predicted death count on day t; α is the intercept; DOM is the dummy variable for day of the week, β is a coefficient in the regression model, X_t_ is the concentration of pollutants on day t; s is the natural cubic spline function; Z_t_ is the meteorological data on day t and df is degree of freedom.

In the model, s (time, df) and s (Z_t_, df) denote the smoothing spline function for nonlinear variables which represent the calendar time and meteorological factor (daily average temperature, air pressure, wind speed, relative humidity) on day t, respectively. The df was selected according to the minimum Akaike Information Criterion. We applied single-air pollutant models to examine the effects of SO_2_/CO on IHD and non-accidental deaths. Meanwhile, we assessed the lag effects of both single-day (distributed lag: lag0-lag5) and multiple-day moving averages (moving average lag: lag01-lag05). In the single-day lag models, lag0-lag5 mean the concentrations of air pollutants of the current day (lag0) and the previous several days (from lag1 to lag5). In multi-day lag models, lag05, for example, means a 6-day moving average pollutant. The two-air pollutant model was used to test the effect of one pollutant on IHD and non-accidental deaths with the other pollutant adjusted in the model. The lag effects of both single-day and multiple-day moving averages were also assessed.

**Fig 2:**
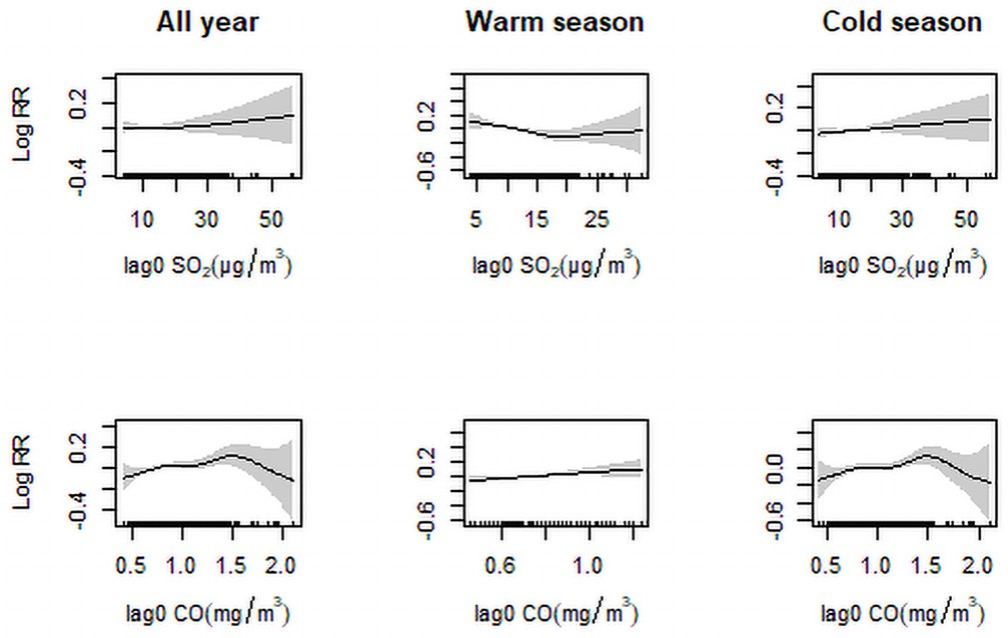
Concentration-response curves (smoothing by natural cubic spline functions with seven degrees of freedom) between levels of sulfur dioxide (SO_2_) and carbon monoxide (CO) and relative risk (RR) of death from ischemic heart disease.

The results are expressed as the relative risk (RR) of IHD and non-accidental deaths for every 10 ug/m^3^ or 10 mg/m^3^ increase in the concentrations of SO_2_ and CO, respectively. The exposure relationships between SO_2_/CO concentrations in different seasons and the log-relative risk of mortality were examined graphically by replacing the linear term of the SO_2_/CO concentration with a smoothing function. The predicted values of the smoothing term, i.e. log-RR, were then plotted against the SO_2_/CO concentrations (Fig 2).

Subgroup analyses were conducted for age (<65 and ≥65 years), sex (male and female), season (May to October [warm] and November to April [cold]) [9, 26, 27] SPSS 19.0 software (IBM SPSS, Armonk, NY, USA) was used for data input, and R statistical software (version 3.2.3, R Foundation for Statistical Computing, Vienna, Australia) was used for data analysis. *P*<0.05 was considered as statistically significant.

## 3. Results

### 3.1 Descriptive statistics

Table 1 shows the descriptive statistics of daily deaths, meteorological data, and air pollutant levels between January 1, 2016 and December 31, 2018. During the study period (1097 days), the total number of the IHD and non-accidental deaths was 10507 and 44070, respectively (9 IHD and 40 non-accidental deaths per day). The IHD daily death counts were higher in males and the elderly (≥65 years) compared with the females and the people less than 65 years. Average daily concentrations of SO_2_ ranged from 3.80 to 57.00 μg/m^3^ (mean: 12.96 μg/m^3^). Average daily concentrations of CO ranged from 0.42 to 2.10 mg/m^3^ (mean: 0.85 mg/m^3^). The concentration of the two pollutants was higher during the cold season (*P* < 0.05). The average air pressure, temperature, relative humidity, and wind speed were 1001.33 ± 8.87 h Pa, 17.64 ± 8.73 °C, 79.27 ± 14.22%, and 2.63 ± 1.43 m/s, respectively.

**Table 1:** Daily deaths, air pollutant levels, and meteorological data in Changsha, between 2016 and 2018.

	Mean	SD	Min	Percentiles			Max	Total
				25th	50th	75th		
Daily death counts (N)								
Non-accidental deaths	40.21	10.7	14	33	39	45.25	119	44070
IHD deaths	9.59	3.97	1	7	9	12	38	10507
Death counts by gender (N)								
Male	23.45	6.88	7	19	23	27	67	5654
Female	16.76	5.63	4	13	16	20	52	4853
Death counts by age (N)								
0–65 years	9.20	3.28	0	7	9	11	20	1584
≥65 years	31.01	9.35	11	25	30	35	104	8923
SO_2_ (μg/m^3^)								
All year	12.96	6.66	3.80	8.20	11.80	16.20	57.00	--
Warm season	11.64	4.86	4.00	8.00	10.60	14.40	32.40	--
Cold season	14.30	7.86	3.80	8.80	13.00	17.60	57.00	--
CO (mg/m^3^)								
All year	0.85	0.23	0.42	0.68	0.80	0.98	2.10	--
Warm season	0.74	0.14	0.46	0.64	0.72	0.82	1.24	--
Cold season	0.96	0.25	0.42	0.80	0.94	1.10	2.10	--
Air pressure (h Pa)								
All year	1001.33	8.87	983.10	993.80	1000.8	1008.00	1029.72	--
Warm season	995.21	6.01	983.10	990.55	994.20	999.13	1015.12	--
Cold season	1007.54	6.71	990.50	1003.07	1007.50	1012.59	1029.72	--
Temperature (°C)								
All year	17.64	8.73	-2.80	10.14	18.30	25.00	32.70	--
Warm season	24.53	4.83	9.75	21.40	24.92	28.41	32.70	--
Cold season	10.66	5.74	-2.80	6.40	10.12	14.90	25.30	--
Relative humidity (%)								
All year	79.27	14.22	35.50	69.5	80.75	91.00	100.00	--
Warm season	80.24	12.01	49.50	71.19	81.00	89.76	100.00	--
Cold season	78.28	16.08	35.50	68.19	80.75	92.76	100.00	--
Wind speed (m/s)								
All year	2.63	1.43	0.00	1.58	2.43	3.54	8.45	--
Warm season	2.52	1.38	0.00	1.50	2.30	3.41	8.45	--
Cold season	2.73	1.48	0.00	1.60	2.52	3.66	7.38	--

IHD, ischemic heart disease; CO, carbon monoxide; Max, maximum; SD, standard deviation; SO_2_, sulfur dioxide; Warm season, May to October; Cold season, November-April.

### 3.2. Spearman correlation

S1 Table shows the daily mean values of the Spearman correlation coefficients between air pollutant levels and meteorological conditions in Changsha during the study period. SO_2_ and CO levels were moderately correlated with each other (0.384, *P* < 0.01). Wind speed was weakly negatively correlated with SO_2_ and CO levels (-0.253 and -0.210 respectively, *P* < 0.01). Air pressure was moderately correlated with CO levels (0.367, *P* < 0.01).

### 3.3. Generalized additive modeling

Table 2 lists RR values and 95% confidence intervals (CIs) of IHD and non-accidental deaths associated with 10-unit increase in pollutant concentrations in lags ranging from zero to 5 days in single-pollutant models. For IHD deaths, the significant RRs observed for SO_2_ were for lag 1–2 and lag 01–05, while for CO, the significant RRs were for lag 0–3 and lag 01–03. The largest significant RR was Lag02 for both SO_2_ (RR: 1.080; 95%CI: 1.075–1.084) and CO (RR: 5.297; 95%CI: 5.177–5.418). The associations of SO_2_ and CO with mortality were more pronounced on moving-average days. Table 3 lists the association of air pollutant levels with IHD and non-accidental death stratified by sex, age group, and season. For IHD deaths, CO levels were associated with death more among men (RR: 5.517; 95%CI: 5.381–5.654) and the elderly (RR: 3.551; 95%CI: 3.439–3.664), and the association was stronger during the warm season (RR: 7.319; 95%CI: 7.708–7.560). However, SO_2_ was statistically significant correlated with the warm season (RR: 0.905; 95%CI: 0.895–0.914).

**Table 2:** Relative risk and 95% confidence intervals of daily deaths associated with a 10-unit increase in the levels of sulfur dioxide (SO_2_) and carbon monoxide (CO) for various lag days in single-pollutant models.

	Lag	SO_2_	CO
Ischemic heart disease deaths	Lag0	1.012(1.008–1.016)	3.256(3.152–3.359)*
	Lag1	1.059(1.055–1.062)**	3.023(2.924–3.121)*
	Lag2	1.051(1.047–1.054)**	3.451(3.352–3.550)*
	Lag3	1.016(1.013–1.020)	0.845(0.743–0.946)
	Lag4	1.020(1.017–1.023)	1.615(1.512–1.718)
	Lag5	1.014(1.011–1.017)	0.680(0.576–0.783)
	Lag01	1.058(1.054–1.063)*	4.012(3.900–4.124)*
	Lag02	1.080(1.075–1.084)**	5.297(5.177–5.418)**
	Lag03	1.074(1.069–1.079)**	3.779(3.650–3.908)*
	Lag04	1.074(1.068–1.079)**	3.821(3.683–3.958)
	Lag05	1.074(1.068–1.080)*	2.925(2.780–3.071)
Non-accidental deaths	Lag0	1.026(1.024–1.028)*	2.977(2.923–3.030)**
	Lag1	1.039(1.037–1.041)**	1.980(1.929–2.031)**
	Lag2	1.032(1.030–1.033)**	1.271(1.221–1.321)
	Lag3	1.009(1.008–1.011)	0.853(0.803–0.903)
	Lag4	0.749(0.698–0.800)	0.993(0.992–0.993)
	Lag5	0.990(0.989–0.992)	0.527(0.476–0.578)*
	Lag01	1.058(1.054–1.063)**	4.012(3.900–4.124)**
	Lag02	1.059(1.057–1.062)**	2.494(2.432–2.557)**
	Lag03	1.053(1.051–1.056)**	1.913(1.847–1.980)
	Lag04	1.043(1.040–1.046)**	1.568(1.498–1.638)
	Lag05	1.034(1.031–1.037)*	1.209(1.135–1.283)

*p < 0.05;

**p < 0.01.

**Table 3:** Association of daily death counts attributable to ischemic heart disease and non-accidental causes with 10-unit increases in concentrations of airborne sulfur dioxide (SO_2_) and carbon monoxide (CO) stratified by age, sex, and season.

		SO_2_		CO	
		Ischemic heart disease deaths	Non-accidental deaths	Ischemic heart disease deaths	Non-accidental deaths
Age group	<65	1.006(0.995–1.016)	1.006(0.995–1.016)	2.008(1.756–2.260)	2.008(1.756–2.260)
	> = 65	1.015(1.011–1.020)	1.022(1.020–1.024)	3.551(3.439–3.664)*	3.072(3.011–3.132)**
Sex	Male	1.029(1.024–1.035)	1.016(1.013–1.019)	5.517(5.381–5.654)*	4.495(4.431–4.559)**
	Female	1.002(0.996–1.008)	1.042(1.039–1.045)*	1.677(1.526–1.828)	1.525(1.441–1.609)
Season	Cold	1.024(1.019–1.029)	1.012(1.009–1.015)	2.742(2.622–2.863)	3.066(3.003–3.129)**
	Warm	0.905(0.895–0.914)*	1.005(1.000–1.009)	7.319(7.708–7.560)	2.972(2.856–3.088)

*p < 0.05;

**p < 0.01.

For non-accidental deaths, the significant RRs observed for SO_2_ were for lag 1–2 and lag 01–05, while for CO, the significant RRs were for lag 0, 1, 5 and lag 01–02. The largest significant RRs in single-pollutant models were lag 02 for SO_2_ [1.059 (95%CI: 1.057–1.062)] and lag 01 for CO [4.012 (95% CI: 3.900–4.124)] in Table 2. SO_2_ levels were associated with non-accidental deaths among the elderly (RR: 1.042; 95%CI: 1.039–1.045); meanwhile, CO levels were associated with death more among men (RR: 4.495; 95%CI: 4.431–4.559), the elderly (RR: 3.072; 95%CI: 3.011–3.132) and the cold season (RR: 3.066; 95%CI: 3.003–3.129) in Table 3.

**Fig 3:**
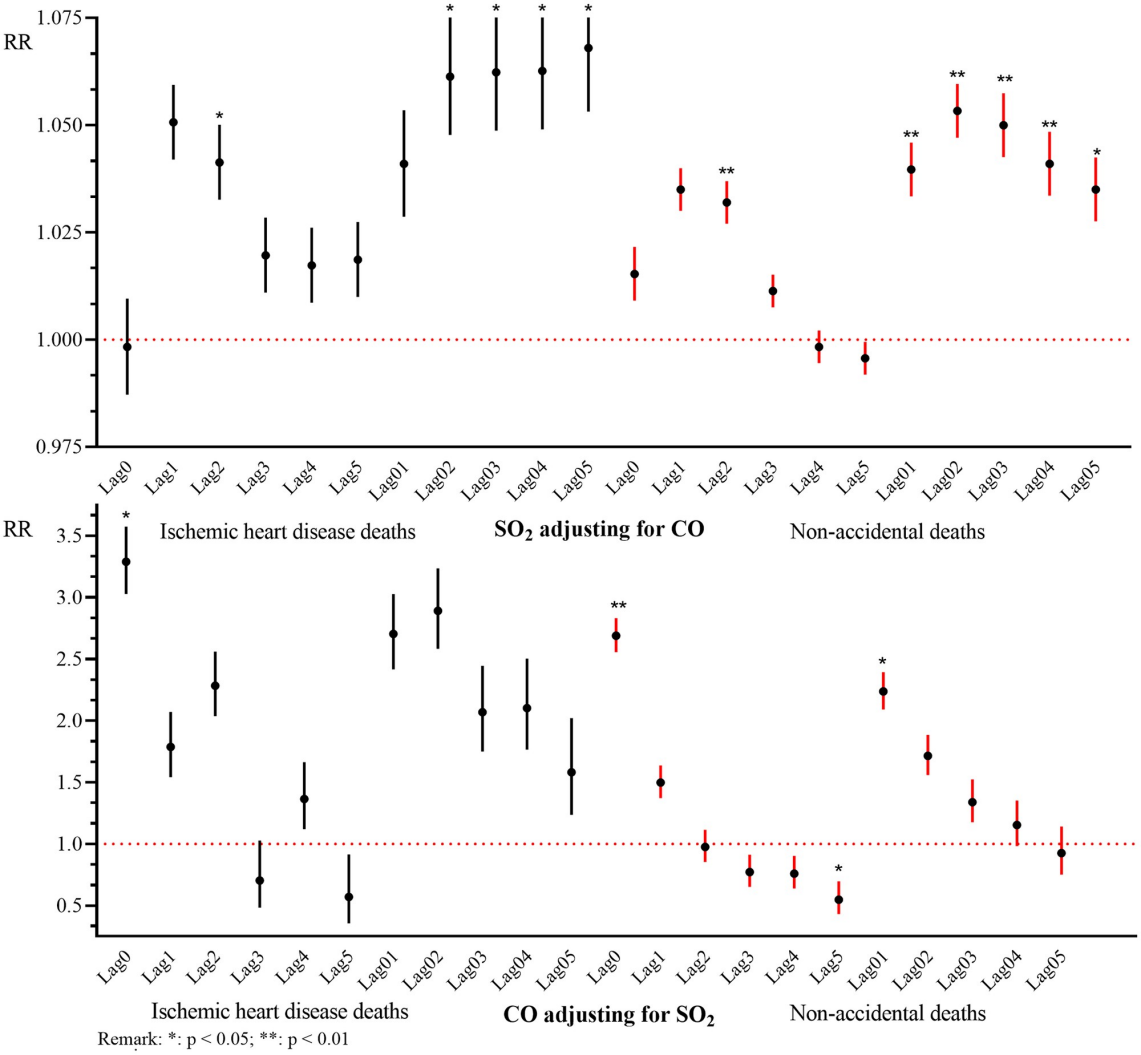
Relative risk (RR) and 95% confidence intervals of daily death counts associated with 10-unit increases in pollutant concentrations for various lags in the two-pollutant model.

Fig 2 presents the exposure-response relationships between air pollutant levels and same-day deaths from IHD in the single-pollutant models during the warm season, the cold season, and annually. The exposure-response curves associated with SO_2_ both during the cold season and annually and with CO during the warm season exhibited similar positive linear relationships. IHD deaths increased notably with incremental additions of CO levels, especially when exposure concentrations were low. Other descending-shaped curves tended to become nonlinear at higher CO concentrations during the cold season and annually. Fig 3 shows the association between pollutant concentrations and daily death counts in the two-pollutant models. For IHD deaths, SO_2_ increased risk significantly from lag 02 to lag 05 but decreased it from lag 1 to lag 4, when CO was included in the model. CO did not increase IHD deaths risk only at lag 3 and lag5 when adjusting for SO_2_. For non-accidental deaths, SO_2_ and CO decreased its risk significantly from lag 1–5 and lag 02–05, when adjusting for the other air pollutant in the model.

## 4. Discussion

In this population-based time-series study, we identified 10,507 IHD and 44,070 non-accidental deaths in Changsha between 2016 and 2018. CO levels were associated with mortality to a greater extent than SO_2_, especially for IHD. Our findings suggest that the association of SO_2_ with death from IHD is greater during the cold season and that of CO is greater during the warm season. In addition, the risk was greater in the elderly for both pollutants and in men for CO. Conversely, the risk of overall non-accidental death attributable to SO_2_ levels was more robust in women. To the best of our knowledge, this is the first comprehensive study of the acute effect of both SO_2_ and CO on mortality from IHD in China.

The main sources of SO_2_ and CO in Changsha come from vehicle exhaust, dining fumes, coal-fired heating, and industrial emissions. SO_2_ and CO levels in Changsha are higher during the cold season, when use of coal for energy is more frequent. Mean levels of SO_2_ and CO in our study were higher than those reported in Wuhan, China and in developed nations [5, 28, 29], but lower than those reported in Shenzhen [30], Beijing [31], and Iran [32]. Nowadays, the China government has been working hard to tackle air pollution by coming up with policies and measures when founding the economic development has been achieved at the expense of the environment. Therefore, the concentration of all air pollutions is starting to decrease [33].

We found lagged associations between SO_2_ and CO with IHD and non-accidental deaths. For each10-mg/m^3^ increase in CO, the RR peaked at a lag of 0–2 days. A nationwide time-series analysis in 272 major cities in China also found that associations between short-term exposure to CO and cardiovascular disease mortality were strongest during the first two lag days [21]. Our results are also similar to a previous estimate in a project conducted in 19 European cities reporting that the 2-day mean CO concentration was associated with an increase in cardiovascular disease mortality [22]. We found that the maximal impact of SO_2_ occurred at a lag of 0–5 days for IHD deaths and 0–2 days for non-accidental deaths. A study in Hong Kong reported a positive correlation between SO_2_ levels and IHD death after a 1-day lag [34], while a study in Wuhan estimated that SO_2_ was associated with non-accidental deaths at lag0 and lag1 [27]. The cumulative lag effects of SO_2_ and CO exhibited increasing trends for both IHD and non-accidental deaths. Investigating the associations between air pollution and disease mortality after delays may clarify their relationships and suggest the disease prevention advice [32].

The curves for SO_2_ to IHD deaths in this study indicated that ambient SO_2_ increased IHD deaths slowly during the cold season but inversely during the warm season, which was confirmed in the stratified analyses. These findings are concordant with published reports of seasonal variations in mortality [35]. SO_2_ effect estimates were more pronounced during the cold season in five urban districts of Changzhou, China [9], but another study reported that SO_2_ was linked to deaths from atherosclerotic heart disease during the warm seasons in Canada [36]. This shows that effects vary by study design, medical condition, pollutant level, and the presence of other pollutants. Temporal variations of vasoconstriction and higher cholesterol and triglyceride concentrations during the cold period could change mortality rates [37]. What is more, the presence of pathogens that cause respiratory infections may also be relevant to the seasonal variation of mortality [38].

We found that ambient CO levels were associated with slow increases in IHD deaths during the warm season; during the cold season, the association between CO levels and IHD mortality first increased and then decreased. Overall, low levels of CO appeared to contribute to IHD deaths while high levels appeared to decrease at high IHD deaths. A study conducted in 126 US urban counties between 1999 and 2005 also found evidence of an association between short-term exposure to ambient CO and risk of IHD hospitalizations well below current US regulatory standards [23]. At low levels, CO causes tissue hypoxia resulting from the binding of CO to hemoglobin in the blood [39]. Reduced oxygen-carrying capacity of hemoglobin predisposes toward cardiac ischemia in persons with coronary artery disease, and exacerbation of cardiovascular symptoms in persons with coronary heart or lung disease. However, we did not found the association between high levels of CO and adverse health effects reported in other studies [40]. A systematic review and meta-analysis based on 2,748 articles published between 1948 and 2014 observed the strongest association ambient CO concentration and mortality in studies from low- and middle-income countries [41]. Pathways other than carboxyhemoglobin formation may cause the toxicity of CO, which is produced by incomplete combustion of hydrocarbons. In the urban areas, the largest source is vehicle exhaust emission, followed by industrial process, central heating and fires. CO concentrations are temporally heterogeneous, with lower concentrations and a greater influence on IHD deaths observed during the warm season, indicating that lower-level CO pollution may actually increase the public health burden. Residents of cities with warmer climate may engage in more outdoor activities, promoting individual exposure to CO. However, one study reported that a 10-mg/m^3^ increase in CO would increase IHD mortality by 21.1% after a 1-day lag [11].

We found a significant effect of SO_2_ and CO on IHD and non-accidental deaths for all ages, but the association was stronger for the elderly (≥65 years), a known high-risk population [27, 42]. Higher risk in the elderly may be attributable to cumulative toxic effects from long-term exposure to ambient pollution and to concomitant cardiovascular morbidity [36]. We found that men were more at risk for IHD and non-accidental deaths associated with CO exposure than women, perhaps because men are more likely to work outdoors [43]. Conversely, women were more at risk for non-accidental deaths associated with SO_2_ exposure, consistent with reports that SO_2_ was only associated with non-accidental deaths in women [27, 44]. We could not identify the mechanism or underlying factors responsible for the sex disparity using a time-series analysis. Therefore, a more accurate exposure assessment or toxicological studies design should be leveraged to explain this phenomenon.

The study showed the association of SO_2_ with deaths did not weaken after adjusting for CO levels. A multicenter European study conducted in seven countries reported that the association of SO_2_ with IHD in individuals aged < 65 years was not modified by CO [14]. However, a study conducted in five urban districts of Changzhou City, China, showed an association between SO_2_ levels and ischemic stroke mortality when adjusting for CO in two-pollutant models [9]. In the study of Wang et al., they reported that the association of SO_2_ exposure with mortality was robust when adjusting for CO [13]. This suggests that SO_2_ may play an independent role in triggering ischemic cardiac events. In contrast, the association between CO levels and mortality was modified by SO_2_, indicating the collinearity of the two pollutants but also the persistence of the SO_2_ effect on mortality. In China, a study conducted in four cities located in the Pearl River Delta observed strong confounding effects of particulate matter and nitrogen dioxide on cardiovascular mortality, in association with exposure to ambient CO [39]. Consistent with our findings, some studies reported that CO was associated with a decreased or non-significant risk of death after adjusting for the other pollutants [45, 46]. However, a study conducted in the 26 largest cities in China showed that the associations of CO with daily hospital admissions for all-cause and cardiovascular diseases were significantly robust to adjustment for co-pollutants [40], perhaps because of a combined effect of gaseous pollutants on cardio-cerebrovascular disease. Exposure to ambient levels of CO, SO_2_, and other pollutants may strengthen or weaken their individual effects. Therefore, toxicological and population studies of exposure to air pollution mixtures may identify the biological mechanisms through which pollutants exert adverse health effects.

However, the limitations in our exposure assessment protocol should be noted in interpreting the results. Firstly, we relied on routine measurements from 10 fixed-site monitoring stations instead of more accurate measurement based on the individuals’ residence. Thus, we were not able to quantify the exposure of individuals precisely. This may lead to errors in the exposure measurement and underestimation of the effects. Second, we did not consider CO/SO_2_ exposure indoor sources. For us, no indoor air monitoring data is available, and future studies may consider that studying the effect of indoor air pollution on IHD deaths or the proportion of indoor air pollution on mortality in the overall air pollution effect. Thirdly, due to the ecological study design which is the use of aggregated data, concerns regarding residual confounding and ecological fallacy remain. Therefore, the results of this study cannot be generalized to the individual level and need to be interpreted as providing an etiological hypothesis. Moreover, the data regarding IHD cases and air pollutant levels were collected from only one city, and it is difficult to extrapolate the results to other areas in China. Further national or multicity studies are required.

The present study also has strengths. First, this study is one of few to report an association between SO_2_ and CO and IHD in a provincial capital city exposure to slight to moderate levels of pollution. Second, our study consisted of a large sample of 10,507 and 44,070 IHD and non-accidental deaths, respectively, and there were no missing data meteorological and air pollution data for the study period, strongly supporting the statistical findings. Finally, the data were acquired from reliable sources and the weather monitoring stations covered the entire urban area of Changsha, representing the pollution situation well. The findings of this study can provide evidence to explore the health risks associated with low concentrations exposure of SO_2_ and CO in south-central China.

## 5. Conclusions

In conclusion, we observed significant associations between short-term exposure to relatively low SO_2_ and CO and increased daily risk of IHD and non-accidental death in the urban districts of Changsha city. Short-term exposure in the cold season to SO_2_ and warm season to CO showed increased concentration-dependent associations with daily IHD deaths. The men have greater risk of death for CO exposure, while the women for SO_2_, and the elderly for both pollutants. Our study provides evidence to explore the health risks associated with low concentrations exposure of SO_2_ and CO in China.

## Supporting information

S1 Table Spearman’s rank correlation between air pollutants and meteorological factors in Changsha, China (2016–2018). (DOCX)

## Abbreviations

CI: confidence interval
CO: carbon monoxide
ICD-10: International Classification of Diseases, 10th Edition
IHD: ischemic heart disease
RR: relative risk
SO_2_: sulfur dioxide

## References

[1] Roth GA, Abate D, Abate KH, Abay SM, Abbafati C, Abbasi N, et al. Global, regional, and national age-sex-specific mortality for 282 causes of death in 195 countries and territories, 1980–2017: a systematic analysis for the Global Burden of Disease Study 2017. The Lancet. 2018; 392:1736–88. 10.1016/S0140-6736(18)32203-7 PMC6227606 30496103

[2] Wang H, Naghavi M, Allen C, Barber RM, Bhutta ZA, Carter A, et al. Global, regional, and national life expectancy, all-cause mortality, and cause-specific mortality for 249 causes of death, 1980–2015: a systematic analysis for the Global Burden of Disease Study 2015. The Lancet. 2016; 388:1459–544. 10.1016/S0140-6736(16)31012-1 PMC5388903 27733281

[3] Xu A, Mu Z, Jiang B, Wang W, Yu H, Zhang L, et al. Acute Effects of Particulate Air Pollution on Ischemic Heart Disease Hospitalizations in Shanghai, China. Int J Environ Res Public Health. 2017; 14. 10.3390/ijerph14020168 28208759 PMC5334722

[4] Chen ZF, Young L, Yu CH, Shiao SPK. A Meta-Prediction of Methylenetetrahydrofolate-Reductase Polymorphisms and Air Pollution Increased the Risk of Ischemic Heart Diseases Worldwide. Int J Environ Res Public Health. 2018; 15. 10.3390/ijerph15071453 29996520 PMC6068673

[5] Dai X, Liu H, Chen D, Zhang J. Association between ambient particulate matter concentrations and hospitalization for ischemic heart disease (I20-I25, ICD-10) in China: A multicity case-crossover study. Atmospheric Environment. 2018; 186:129–35.

[6] Tam W, Wong TW, Wong A. Association between air pollution and daily mortality and hospital admission due to Ischaemic heart diseases in Hong Kong. Atmospheric Environment. 2015; 120.

[7] WHO. Air pollution. 2020; https://www.who.int/health-topics/air-pollution.

[8] WHO. Ambient air pollution: Health impacts. 2016; https://www.who.int/airpollution/ambient/health-impacts/en/.

[9] Dong H, Yu Y, Yao S, Lu Y, Chen Z, Li G, et al. Acute effects of air pollution on ischaemic stroke onset and deaths: a time-series study in Changzhou, China. BMJ Open. 2018; 8:e020425. 10.1136/bmjopen-2017-020425 30037864 PMC6059268

[10] Wu Z, Li J, Huang J, Wang Y, Cao R, Yin P, et al. Ambient sulfur dioxide and years of life lost from stroke in China: a time-series analysis in 48 cities. Chemosphere. 2020:128857. 10.1016/j.chemosphere.2020.128857 33183785

[11] Dastoorpoor M, Riahi A, Yazdaninejhad H, Borsi SH, Khanjani N, Khodadadi N, et al. Exposure to particulate matter and carbon monoxide and cause-specific Cardiovascular-Respiratory disease mortality in Ahvaz. Toxin Reviews. 2020:1–11.

[12] Deng Q, Lu C, Jiang W, Zhao J, Deng L, Xiang Y. Association of outdoor air pollution and indoor renovation with early childhood ear infection in China. Chemosphere. 2017; 169:288–96. 10.1016/j.chemosphere.2016.11.079 27883914

[13] Wang L, Liu C, Meng X, Niu Y, Lin Z, Liu Y, et al. Associations between short-term exposure to ambient sulfur dioxide and increased cause-specific mortality in 272 Chinese cities. Environment International. 2018; 117:33–9. 10.1016/j.envint.2018.04.019 29715611

[14] Sunyer J. The association of daily sulfur dioxide air pollution levels with hospital admissions for cardiovascular diseases in Europe (The Aphea-II study). European Heart Journal. 2003; 24:752–60. 10.1016/s0195-668x(02)00808-4 12713769

[15] Wang DZ, Jiang GH, Zhang H, Song GD, Zhang Y. [Effect of air pollution on coronary heart disease mortality in Tianjin, 2001–2009: a time-series study]. Zhonghua liu xing bing xue za zhi = Zhonghua liuxingbingxue zazhi. 2013; 34:478–83. 24016439

[16] Liu Y, Chen X, Huang S, Tian L, Lu Y, Mei Y, et al. Association between air pollutants and cardiovascular disease mortality in Wuhan, China. Int J Environ Res Public Health. 2015; 12:3506–16. 10.3390/ijerph120403506 25815523 PMC4410199

[17] Protection MoE. China Environmental Statements Bulletin2015.

[18] Kharol SK, Mclinden CA, Sioris CE, Shephard MW, Martin RVJAC, Physics. OMI satellite observations of decadal changes in ground-level sulfur dioxide over North America. 2016:1–17.

[19] Protection MoEaE. China Ecological Environment Status Bulletin. 2019.

[20] Tirosh E, Schnell I. The relationship between ambient carbon monoxide and heart rate variability-a systematic world review-2015. Environ Sci Pollut Res Int. 2016; 23:21157–64. 10.1007/s11356-016-7533-0 27623853

[21] Liu C, Yin P, Chen R, Meng X, Wang L, Niu Y, et al. Ambient carbon monoxide and cardiovascular mortality: a nationwide time-series analysis in 272 cities in China. The Lancet Planetary Health. 2018; 2:e12–e8. 10.1016/S2542-5196(17)30181-X 29615203

[22] Samoli E, Touloumi G, Schwartz J, Anderson HR, Schindler C, Forsberg B, et al. Short-term effects of carbon monoxide on mortality: an analysis within the APHEA project. Environ Health Perspect. 2007; 115:1578–83. 10.1289/ehp.10375 18007988 PMC2072841

[23] Bell ML, Peng RD, Dominici F, Samet JM. Emergency hospital admissions for cardiovascular diseases and ambient levels of carbon monoxide: results for 126 United States urban counties, 1999–2005. Circulation. 2009; 120:949–55. 10.1161/CIRCULATIONAHA.109.851113 19720933 PMC2777712

[24] Lei R, Zhu F, Cheng H, Liu J, Shen C, Zhang C, et al. Short-term effect of PM2.5/O3 on non-accidental and respiratory deaths in highly polluted area of China. Atmospheric Pollution Research. 2019; 10:1412–9.

[25] Kan H, London SJ, Chen G, Zhang Y, Song G, Zhao N, et al. Season, sex, age, and education as modifiers of the effects of outdoor air pollution on daily mortality in Shanghai, China: The Public Health and Air Pollution in Asia (PAPA) Study. Environ Health Perspect. 2008; 116:1183–8. 10.1289/ehp.10851 18795161 PMC2535620

[26] Zhang C, Ding R, Xiao C, Xu Y, Cheng H, Zhu F, et al. Association between air pollution and cardiovascular mortality in Hefei, China: A time-series analysis. Environ Pollut. 2017; 229:790–7. 10.1016/j.envpol.2017.06.022 28797522

[27] Zhong P, Huang S, Zhang X, Wu S, Zhu Y, Li Y, et al. Individual-level modifiers of the acute effects of air pollution on mortality in Wuhan, China. Glob Health Res Policy. 2018; 3:27. 10.1186/s41256-018-0080-0 30214944 PMC6131956

[28] Ren M, Li N, Wang Z, Liu Y, Chen X, Chu Y, et al. The short-term effects of air pollutants on respiratory disease mortality in Wuhan, China: comparison of time-series and case-crossover analyses. Sci Rep. 2017; 7:40482. 10.1038/srep40482 28084399 PMC5234024

[29] Roye D, Zarrabeitia MT, Riancho J, Santurtun A. A time series analysis of the relationship between apparent temperature, air pollutants and ischemic stroke in Madrid, Spain. Environ Res. 2019; 173:349–58. 10.1016/j.envres.2019.03.065 30953949

[30] Cai J, Yu S, Pei Y, Peng C, Liao Y, Liu N, et al. Association between Airborne Fine Particulate Matter and Residents’ Cardiovascular Diseases, Ischemic Heart Disease and Cerebral Vascular Disease Mortality in Areas with Lighter Air Pollution in China. Int J Environ Res Public Health. 2018; 15. 10.3390/ijerph15091918 30177663 PMC6164472

[31] Li W, Cao Y, Li R, Ma X, Chen J, Wu Z, et al. The spatial variation in the effects of air pollution on cardiovascular mortality in Beijing, China. J Expo Sci Environ Epidemiol. 2018; 28:297–304. 10.1038/jes.2016.21 29666509

[32] Dastoorpoor M, Goudarzi G, Khanjani N, Idani E, Aghababaeian H, Bahrampour A. Lag time structure of cardiovascular deaths attributed to ambient air pollutants in Ahvaz, Iran, 2008–2015. Int J Occup Med Environ Health. 2018; 31:459–73. 10.13075/ijomeh.1896.01104 29546882

[33] Xiong L, Xu Z, Tan J, Wang H, Liu Z, Wang A, et al. Acute effects of air pollutants on adverse birth outcomes in Changsha, China: A population data with time-series analysis from 2015 to 2017. Medicine (Baltimore). 2019; 98:e14127. 10.1097/MD.0000000000014127 30653143 PMC6370066

[34] Wong TW, Tam WS, Yu TS, Wong AH. Associations between daily mortalities from respiratory and cardiovascular diseases and air pollution in Hong Kong, China. Occupational and environmental medicine. 2002; 59:30–5. 10.1136/oem.59.1.30 11836466 PMC1740206

[35] Ha S, Nguyen K, Liu D, Mnnist T, Nobles C, Sherman S, et al. Ambient temperature and risk of cardiovascular events at labor and delivery: a case-crossover study. 2017; 159. 10.1016/j.envres.2017.09.010 PMC5624535 28926807

[36] Goldberg MS, Burnett RT, Stieb DM, Brophy JM, Daskalopoulou SS, Valois MF, et al. Associations between ambient air pollution and daily mortality among elderly persons in Montreal, Quebec. Sci Total Environ. 2013; 463–464:931–42. 10.1016/j.scitotenv.2013.06.095 23872247

[37] Ockene IS, Chiriboga DE, Stanek EJ 3rd, Harmatz MG, Nicolosi R, Saperia G, et al. Seasonal variation in serum cholesterol levels: treatment implications and possible mechanisms. Archives of internal medicine. 2004; 164:863–70. 10.1001/archinte.164.8.863 15111372

[38] Huang CL, Nguyen PA, Kuo PL, Iqbal U, Hsu YH, Jian WS. Influenza vaccination and reduction in risk of ischemic heart disease among chronic obstructive pulmonary elderly. Comput Methods Programs Biomed. 2013; 111:507–11. 10.1016/j.cmpb.2013.05.006 23769164

[39] Raub JA, Mathieu-Nolf M, Hampson NB, Thom SR. Carbon monoxide poisoning—a public health perspective. Toxicology. 2000; 145:1–14. 10.1016/s0300-483x(99)00217-6 10771127

[40] Liu H, Tian Y, Xiang X, Li M, Wu Y, Cao Y, et al. Association of short-term exposure to ambient carbon monoxide with hospital admissions in China. Scientific Reports. 2018; 8. 10.1038/s41598-018-31434-1 30190544 PMC6127141

[41] Shah AS, Lee KK, McAllister DA, Hunter A, Nair H, Whiteley W, et al. Short term exposure to air pollution and stroke: systematic review and meta-analysis. BMJ (Clinical research ed). 2015; 350:h1295. 10.1136/bmj.h1295 25810496 PMC4373601

[42] Cakmak S, Dales RE, Rubio MA, Vidal CB. The risk of dying on days of higher air pollution among the socially disadvantaged elderly. Environ Res. 2011; 111:388–93. 10.1016/j.envres.2011.01.003 21256481

[43] Qiu H, Yu IT, Wang X, Tian L, Tse LA, Wong TW. Cool and dry weather enhances the effects of air pollution on emergency IHD hospital admissions. International journal of cardiology. 2013; 168:500–5. 10.1016/j.ijcard.2012.09.199 23079091

[44] Liu G, Sun B, Yu L, Chen J, Han B, Liu B, et al. Short-term exposure to ambient air pollution and daily atherosclerotic heart disease mortality in a cool climate. Environ Sci Pollut Res Int. 2019; 26:23603–14. 10.1007/s11356-019-05565-5 31203548

[45] Chen R, Pan G, Zhang Y, Xu Q, Zeng G, Xu X, et al. Ambient carbon monoxide and daily mortality in three Chinese cities: the China Air Pollution and Health Effects Study (CAPES). Sci Total Environ. 2011; 409:4923–8. 10.1016/j.scitotenv.2011.08.029 21908017

[46] Tsai SS, Huang CH, Goggins WB, Wu TN, Yang CY. Relationship between air pollution and daily mortality in a tropical city: Kaohsiung, Taiwan. Journal of toxicology and environmental health Part A. 2003; 66:1341–9. 10.1080/15287390306389 12851115

