# Supplementary material for: Association between short-term exposure to sulfur dioxide and carbon monoxide and ischemic heart disease and non-accidental death in Changsha city, China
Source: PLoS One. 2021 May 3;16(5):e0251108. doi: 10.1371/journal.pone.0251108 (PMC8092655; doi:10.1371/journal.pone.0251108)
Supplement: S1 Table — (DOCX) [file pone.0251108.s001.docx]

**S1 Table. Spearman’s rank correlation between air pollutants and meteorological factors in Changsha, China (2016-2018).**

|  | Air pressure | Temperature | Relative humidity | Wind speed | SO_2_ | CO |
| --- | --- | --- | --- | --- | --- | --- |
| Air pressure | 1.000 |  |  |  |  |  |
| Temperature | -0.906** | 1.000 |  |  |  |  |
| Relative humidity | -0.061* | -0.153** | 1.000 |  |  |  |
| Wind speed | 0.154** | -0.196** | 0.213** | 1.000 |  |  |
| SO_2_ | 0.117** | 0.000 | -0.487** | -0.253** | 1.000 |  |
| CO | 0.367** | -0.421** | 0.058 | -0.210** | 0.384** | 1.000 |

**p* < 0.05; ***p* < 0.01. CO, carbon monoxide; SO_2_, sulfur dioxide.
